# A Bayesian space–time model for clustering areal units based on their disease trends

**DOI:** 10.1093/biostatistics/kxy024

**Published:** 2018-06-18

**Authors:** Gary Napier, Duncan Lee, Chris Robertson, Andrew Lawson

**Affiliations:** 1 School of Mathematics and Statistics, University of Glasgow, University Place, Glasgow, UK; 2 Department of Mathematics and Statistics, University of Strathclyde, 26 Richmond Street, Glasgow, UK; 3 Department of Public Health Sciences, Medical University of South Carolina, South Carolina, USA

**Keywords:** Health inequalities, Metropolis-Coupled Markov chain Monte Carlo ((MC)^3^) simulation, Space-time disease mapping, Trend estimation

## Abstract

Population-level disease risk across a set of non-overlapping areal units varies in space and time, and a large research literature has developed methodology for identifying clusters of areal units exhibiting elevated risks. However, almost no research has extended the clustering paradigm to identify groups of areal units exhibiting similar temporal disease trends. We present a novel Bayesian hierarchical mixture model for achieving this goal, with inference based on a Metropolis-coupled Markov chain Monte Carlo ((MC)$^3$) algorithm. The effectiveness of the (MC)$^3$ algorithm compared to a standard Markov chain Monte Carlo implementation is demonstrated in a simulation study, and the methodology is motivated by two important case studies in the United Kingdom. The first concerns the impact on measles susceptibility of the discredited paper linking the measles, mumps, and rubella vaccination to an increased risk of Autism and investigates whether all areas in the Scotland were equally affected. The second concerns respiratory hospitalizations and investigates over a 10 year period which parts of Glasgow have shown increased, decreased, and no change in risk.

## 1. Introduction

Population-level disease risk varies between communities due to variation in factors such as air pollution concentrations and smoking rates, while temporal trends can be affected by public health interventions and health scares. Spatial variation in disease risk is known as a health inequality, with more affluent communities typically exhibiting lower disease risks compared to more impoverished ones (Mackenbach *and others*, 1997). Health inequalities are recognized internationally as a key public health challenge (World Health Organisation, 2013) and have gained political traction in the United Kingdom following the Marmot review (Marmot, 2010). They can be quantified by modeling small-area population-level disease incidence data, which enables policy-relevant questions to be answered, such as: which areas exhibit elevated risks and increased risk trends compared to their geographical neighbors; and, does a health scare have the same impact on disease risk in all socio-economic groups in society? A wide range of statistical models have been proposed for modeling spatio-temporal variation in disease risk, with the most popular models including those proposed by Bernardinelli *and others* (1995) and Knorr-Held (2000).

One popular goal in modeling small-area disease data is the identification of clusters of areas exhibiting elevated risks compared with their geographical neighbors, and a number of approaches have been proposed including Knorr-Held and Rasser (2000), Charras-Garrido *and others* (2013), and Wakefield and Kim (2013) in a spatial context, while Lee and Lawson (2016) extended this to the spatio-temporal setting. While a number of models have been developed for estimating area specific temporal trends (e.g. Bernardinelli *and others*, 1995; MacNab and Dean, 2001), little research has extended the clustering paradigm to group areas together that exhibit similar temporal risk trends. Heard *and others* (2006), Lawson *and others* (2010), Choi *and others* (2011), and Jiang and Serban (2012) propose clustering methodology to group areas together based on sharing common latent temporal trends, but the resulting trends are not shape constrained, as they are represented by spline basis functions or autoregressive processes. Therefore, two or more of the estimated trend functions could be similar, resulting in two areas from different trend clusters exhibiting very similar trends. At the other extreme, Anderson *and others* (2016) augment the linear trends model of Bernardinelli *and others* (1995) with a clustering mechanism, but this restricts all the trends to be linear.

Therefore, we propose a novel Bayesian spatio-temporal mixture model for clustering areas based on their temporal trends, where the candidate trend functions have fixed parametric forms (e.g. linear, step-change) or constrained shapes (e.g. monotonically increasing). The use of fixed or shape-constrained trends is beneficial for two reasons. Firstly, it allows the user to test specific hypotheses about the data being modeled, for example by including a change point trend to determine which areas were affected by a health scare. Secondly, by choosing different shapes for the candidate trends it overcomes the identifiability problem of areas in two supposedly different clusters actually having very similar estimated trends. The choice of the trend functions will be problem specific, and will depend on the temporal patterns in the data, *a priori* knowledge about the context of the data, and the goals of the analysis. Our model is presented in Section 3, while its clustering performance is assessed by simulation in Section 4. The methodology is motivated by two important public health case studies in the United Kingdom, namely measles susceptibility and respiratory hospitalizations, which are presented in Sections 2 (exploratory analysis) and 5 (results). Finally, Section 6 concludes the article.

## 2. Motivating case studies

Our methodology is motivated by two important public health case studies in the United Kingdom, and in both cases our goal is to cluster areas together that share common disease risk trends. Thus, we do not include any covariates in the modeling as then any trends would be in the residual risk after covariate adjustment.

### 2.1. Case study 1—measles susceptibility in Scotland

In 1998, Wakefield *and others* (1998) linked the measles, mumps, and rubella (MMR) vaccine with an increased risk of autism, and the resulting media coverage resulted in vaccination rates dropping to around 80% in 2003 in parts of the United Kingdom (McIntyre and Leask, 2008). These reduced rates were felt in 2013, when a large outbreak of measles occurred in the United Kingdom (Pollock *and others*, 2014). The article by Wakefield *and others* (1998) was partially retracted in 2004, before being fully discredited in 2010 after multiple epidemiological studies failed to find any association with an increased risk of autism (e.g. Elliman and Bedford, 2007).

The MMR vaccine was introduced in Scotland in 1998, and vaccination records were recorded in the Scottish Immunisation & Recall System (SIRS). The data were provided by Health Protection Scotland and relate to children eligible to attend pre-school (aged between 2.5 and 4.5 years) from non-overlapping 2-year birth cohorts between 1998 and 2014. Thus, we have data for $N=9$ time periods (2-years apart) for the set of $K=1235$ intermediate zones (IZ) across Scotland (average population around 4000), which is an administrative geography for distributing small-area statistics. The data comprise the number of children susceptible to measles $Y_{kt}$, and the total number of children $N_{kt}$, for the $k$th IZ and $t$th time period. An exploratory measure of risk is the proportion susceptible $\hat{\theta}_{kt} = Y_{kt}/N_{kt}$, where susceptibility is based on the receipt of one or two vaccinations that each have a 10% failure rate.

The spatial patterns and temporal trends in $\{\hat{\theta}_{kt}\}$ are displayed in Appendix A of the supplementary material available at *Biostatistics* Online, and temporally there appears to be an increase in susceptibility between 1998 and 2004 before a decrease in subsequent years. The existence of such a change point in 2004 is reinforced by our prior knowledge about the date of the partial retraction of the Wakefield paper, as well as the work of Napier *and others* (2016) which showed that the Scotland-wide average trend also contained a change point in 2004. Here, we extend that earlier analysis and estimate whether: (i) all IZs exhibited a change point in measles susceptibility or whether some showed no effect of the articles’ retraction; and (ii) did the change point occur in 2004 for all IZs, or was it earlier or later for some? Therefore, we consider two types of candidate trends in the modeling, linear (increasing, decreasing, and constant) trends indicating no change point, and change point trends with different times for the change point.

### 2.2. Case study 2—Respiratory hospital admissions

Respiratory disease is the second most common cause of death in Scotland behind cancer (http://www.gov.scot/Topics/Statistics/Browse/Health/TrendMortalityRates), and in this study, we focus on the Greater Glasgow and Clyde health board because Glasgow is one of the unhealthiest cities in Europe (Gray *and others*, 2012). We have yearly data for $N=10$ years between 2002 and 2011 for the $K=271$ IZ that make up the health board. For the $k$th IZ and $t$th year, $Y_{kt}$ denotes the number of hospital admissions with a primary diagnosis of respiratory disease (International classification of disease 10th revision codes J00–J99), but this depends on the size and demographic structure of the population in each IZ and year. This is accounted for by computing the expected number of admissions $E_{kt}$ using indirect standardization, based on national age and sex specific hospitalization rates. An exploratory measure of disease risk is the standardized morbidity ratio (SMR) computed as $\hat{\theta}_{kt} = Y_{kt} / E_{kt}$, where a value of 1.2 corresponds to a 20% elevated risk compared to the Scottish average.

The spatial pattern and temporal trends in SMR are displayed in Appendix A of the supplementary material available at *Biostatistics* Online, and the latter again highlights what types of trends are likely to be present in the data. The temporal trend figure shows no clear temporal trends, as some IZs show an increased risk while others show a decreased risk. The magnitude of the health inequalities appears to change little over the 10 year period, as the variation in the SMR in 2002 is similar to that in 2011, with standard deviations of 0.33 and 0.31, respectively. Our key motivating questions for these data are: (i) which areas exhibit an increase, a decrease, or no change in risk over the 10 year period and (ii) how have these changes in risk impacted upon health inequalities. To answer these questions, we consider three candidate trend functions: an increasing trend, a decreasing trend, and no change, and we compare linear and non-linear trends and assess the sensitivity of the results to this choice.

## 3. Methodology

We propose a novel spatio-temporal mixture model for clustering areas based on their temporal trends, which differs from space–time risk models such as Knorr-Held (2000) that do not identify any clusters in the data. Inference is based on a Bayesian setting via a Metropolis-coupled Markov chain Monte Carlo (MC)$^3$ algorithm. The model and inferential algorithm are described in Sections 3.1 to 3.3, while other risk models applied to our data are defined in Section 3.4.

### 3.1. Model specification

The study region is partitioned into $k=1,\ldots,K$ areal units, and data are available for $t=1,\ldots,N$ time periods. Letting $(Y_{kt}, O_{kt})$ respectively denote the response variable and offset (e.g. $\ln(E_{kt})$ in case study 2) in area $k$ and time period $t$, the general model is given by:

(3.1) \begin{eqnarray*} \label{Model:LTM} \begin{split} Y_{kt} &\sim p(y_{kt}| \mu_{kt}), k=1,\ldots,K , t=1,\ldots,N, \\ g(\mu_{kt}) &= O_{kt} + \mathbf{x}_{kt}^\top{\boldsymbol{\beta}} + \phi_k + \sum_{s=1}^S \omega_{ks} f_s(t|{\boldsymbol{\gamma}}_s). \end{split} \end{eqnarray*}

The model has a generalized linear model form with data likelihood $p(y_{kt}| \mu_{kt})$, mean $\mu_{kt}$, and link function $g(\mu_{kt})$. In case study 1, the binomial model $Y_{kt} \sim \text{Binomial}(N_{kt}, \theta_{kt})$ with logit link function $g(\theta_{kt})=\ln(\theta_{kt}/(1-\theta_{kt}))$ is appropriate for the non-rare measles susceptibility outcome, while for case study 2, the Poisson model $Y_{kt} \sim \text{Poisson}(E_{kt}\theta_{kt})$ with log link function $g(\theta_{kt})=\ln(\theta_{kt})$ is appropriate for the rare hospitalization outcome. The spatio-temporal pattern in $\mu_{kt}$ is modeled by the offset $O_{kt}$, a $p \times 1$ vector of covariates $\mathbf{x}_{kt}$ (if required) with parameters ${\boldsymbol{\beta}}=(\beta_1,\ldots,\beta_p)$, a spatial component $\phi_k$ common to all time periods, and a clustering model $\sum_{s=1}^S \omega_{ks} f_s(t|{\boldsymbol{\gamma}}_s)$ for assigning each area to one of $S$ temporal trends $(f_1(t|{\boldsymbol{\gamma}}_1),\ldots,f_S(t{\boldsymbol{\gamma}}_S))$. The regression parameters are assigned the weakly informative prior $\beta_j\sim \text{N}(0, 1000)$. As the aim of the model is to cluster areas based on their overall temporal trends, we have not included temporally varying random effects in the model, because this would mean that the trend functions would then capture the residual trends after adjusting for these random effects.

#### 3.1.1. Overall spatial pattern

The spatial pattern in risk ${\boldsymbol{\phi}} = (\phi_1,\ldots,\phi_K)$ common to all time periods is modeled using the conditional autoregressive (CAR) prior proposed by Leroux *and others* (2000), which uses a $K \times K$ neighborhood matrix $\mathbf{W}$ to define spatial closeness. Here element $w_{kj} = 1$ if areas $(k, j)$ share a common border, otherwise $w_{kj} = 0$ and $w_{kk} = 0$$\forall$$k$. This implies that pairs of areas for which $w_{kj}=1$ are modeled as autocorrelated, whilst pairs of areas where $w_{kj}=0$ are modeled as conditionally independent. The CAR prior is given by

(3.2) \begin{align*} \phi_k | {\boldsymbol{\phi}}_{-k}, {\bf{W}}, \rho, \tau^2 \sim \text{N} \left(\frac{\rho \sum_{j =1}^K w_{kj} \phi_j}{\rho \sum_{j=1}^K w_{kj} + 1 - \rho}, \frac{\tau^2}{\rho \sum_{j=1}^K w_{kj} + 1 - \rho} \right),\label{leroux} \end{align*}

where ${\boldsymbol{\phi}}_{-k} = \left(\phi_1,\dots,\phi_{k-1}, \phi_{k+1}, \ldots, \phi_K \right)$. The strength of the spatial autocorrelation is controlled by $\rho$, with $\rho=1$ corresponding to the intrinsic CAR model of Besag *and others* (1991) for strong spatial autocorrelation, while $\rho=0$ corresponds to independence as $\phi_k\sim\mbox{N}(0, \tau^2)$. We assign a uniform prior for $\rho$, that is $\rho \sim \text{Uniform}(0, 1)$, while an Inverse-Gamma prior is placed upon the spatial variance parameter $\tau^2 \sim \text{Inverse-Gamma}(a=1, b=0.1)$, following (https://github.com/stan-dev/stan/wiki/Prior-Choice-Recommendations).

#### 3.1.2. Mixture model for the trends

The model clusters areas according to their temporal trends via the mixture component $\sum_{s=1}^S \omega_{ks} f_s(t|{\boldsymbol{\gamma}}_s)$, where the $S$ trends $(f_1(t|{\boldsymbol{\gamma}}_1),\ldots,f_S(t{\boldsymbol{\gamma}}_S))$ are chosen by the user. Area $k$ is assigned to one of the $S$ candidate trends via the binary indicator variables ${\boldsymbol{\omega}}_k= (\omega_{k1}, \ldots, \omega_{kS})$, where $\omega_{ks}=1$ if area $k$ is assigned to trend $s$ and is zero otherwise, and $\sum_{s=1}^{S}\omega_{ks}=1$ for all $k$. Therefore, we specify the following multinomial prior distribution for ${\boldsymbol{\omega}}_k$:

(3.3) \begin{eqnarray*} {\boldsymbol{\omega}}_k= (\omega_{k1}, \ldots, \omega_{kS}) &\sim& \text{Multinomial}(1; {\boldsymbol{\lambda}}),\label{cluster model}\\ {\boldsymbol{\lambda}} = (\lambda_1,\ldots,\lambda_S)&\sim&\text{Dirichlet}({\boldsymbol{\alpha}}= (\alpha_1, \ldots, \alpha_S)).\nonumber \end{eqnarray*}

The region-wide probabilities associated with each temporal trend are denoted by ${\boldsymbol{\lambda}} = (\lambda_1,\ldots,\lambda_S)$, and a conjugate weakly informative Dirichlet prior distribution is placed upon these probabilities, where $\alpha_i = 1$ for $i=1,\ldots,S$. The trend indicators ${\boldsymbol{\omega}}_k$ are modeled as independent rather than spatially autocorrelated because spatial autocorrelation in the data is already modeled by ${\boldsymbol{\phi}}$, and additionally, we want the data to determine the clustering of the areas to trends rather than *a priori* encouraging geographically neighboring areas to exhibit the same trends. During the (MC)$^{3}$ algorithm different values of ${\boldsymbol{\omega}}_k$ will be sampled, which allows the computation of the posterior probability that each area is assigned to each trend. We use the maximum *a posteriori* rule for classifying an area to one of the $S$ trends (also used by Lawson *and others*, 2012), because it allows a hard classification of an area to a single trend.

### 3.2. Choice of trend functions

The choice of which and how many trend functions to include in the model will be made by the user and depend on a number of considerations, including: the motivating question being addressed; prior knowledge about any events that may induce specific trends into the data [e.g. the retraction of the Wakefield *and others* (1998) article for the MMR example introducing a change point]; and visually examining plots of risk trends in the raw data (e.g. sample proportions or SMRs). One constraint is that the trends included should be different from each other, as we have found that including two similar trends leads to areas swapping between these trends, resulting in a poor cluster identification and label switching. An implication of this is that including two or more unconstrained trends, such as random walks, is inappropriate as their estimated shapes could be similar to each other. Therefore, we recommend either fixed parametric trends or shape-constrained smooth functions such as those proposed by Pya and Wood (2015). Below, we describe the trends we use in the two case studies.

**Case study 1** — Our goal is to determine which IZs exhibit a change point in measles susceptibility, or whether some IZs showed no effect of the articles’ retraction. Therefore, we compare the following linear (no effect) and change point trends.
  (1) Constant: $f(t) = 0$.
  (2) Linear: $f(t|\gamma) = \gamma t$, which via the prior specification can be constrained to be increasing (via $\gamma \sim \text{N}(0, 1000)\mathbb{I}[\gamma > 0]$) or decreasing (via $\gamma \sim \text{N}(0, 1000)\mathbb{I}[\gamma < 0]$), where $\mathbb{I}[.]$ denotes an indicator function.
  (3) Known change point: $f(t|{\boldsymbol{\gamma}}) = \gamma_1 t + \gamma_2(t - t^*)_+$, where $t^*$ denotes the known change point, and $(t - t^*)_+ = t - t^*$ if $t - t^* > 0$, and is zero otherwise. Different shapes can be specified via the choice of prior distribution for $(\gamma_1, \gamma_2)$, and from our exploratory analysis we constrain the trend to increase and then decrease.

Note that (3.1) includes an intercept term $\beta_1$, which is why no intercept terms are included in the trends above. Also, we can include two linear trends in the same model because they are respectively constrained to be increasing and decreasing, whereas including two unconstrained linear trends may lead to the identification problems outlined above.

**Case study 2** — Our goal is to determine which IZs exhibit increasing, decreasing or no change in respiratory hospitalization rates, which motivates the use of three trend functions. Initially, we included a no-change function ((1) from above) and linear increasing and decreasing trends ((2) from above), but we also consider replacing the linear trends with the following more flexible monotonic alternatives.
  (4) Monotonic cubic splines: $f(t|{\boldsymbol{\gamma}}) = \gamma_0 t + \sum_{j=1}^q \gamma_j(t - t_j^*)_+^3$, which unlike the previous examples allows the shape of the trends to be estimated from the data. Here, $q$ is the number of knots and controls the wiggliness of the estimated trend. To ensure monotonicity, the coefficients $(\gamma_0,\gamma_1,\ldots,\gamma_q)$ are constrained to be positive or negative for monotonically increasing and decreasing trends, respectively, using the same half normal priors outlined above.

### 3.3. Inference

Inference in a Bayesian setting is typically based on either Markov chain Monte Carlo (MCMC) simulation or Integrated Nested Laplace Approximations (INLA, Rue *and others*, 2009), and the latter has become increasingly popular in spatio-temporal modeling (see Lawson *and others*, 2014; Lee and Mitchell, 2014) thanks to the R-INLA package and the excellent book by Blangiardo and Cameletti (2015). However, while R-INLA is able to fit a wide range of spatio-temporal models, such as that proposed by Knorr-Held (2000), it is not able to fit the model proposed here which contains a clustering model to group areas together based on shared trends.

Therefore, we initially developed an MCMC simulation algorithm for model fitting using a combination of Gibbs sampling and random walk Metropolis steps. However, this algorithm performed poorly (see Section 4) and often got trapped in a local mode, which is due to the multimodality issues inherent in fitting mixture models in a Bayesian setting using MCMC simulation (see Atchadé *and others*, 2011; Altekar *and others*, 2004). To overcome this, we developed a (MC)$^3$ algorithm, which runs multiple Markov chains in parallel and then couples the chains together to prevent them from becoming stuck in a local rather than a global mode. The parallel chains are run at different ‘temperature’ levels, where the higher the temperature level the more likely a chain is to accept a proposed move, thus potentially making larger jumps between the multiple modes around the target distribution. This is known as parallel tempering. Full details of our (MC)$^{3}$ algorithm are presented in Appendix B of the supplementary material available at *Biostatistics* Online.

Our inferential algorithms are implemented in R and C++ (via the Rcpp package) and exploit computationally efficient mathematical forms such as triplet form for $\mathbf{W}$. Their computational complexities are summarized in Appendix C of the supplementary material available at *Biostatistics* Online for a range of data sizes, which illustrates the scalability of our methodology to large data sets. Software to fit our model together with the respiratory hospitalization data are provided at https://github.com/GNapier/SpaceTimeClusteringDiseaseTrends to make the results reproducible. However, the measles susceptibility data cannot be provided due to the agreement with the data provider.

### 3.4. Competitor models

In the real data studies in Section 5, we compare the model proposed above in terms of overall model fit to two different competitor models. Both models have the general form

(3.4) \begin{eqnarray*} \label{Model:LTM2} \begin{split} Y_{kt} &\sim p(y_{kt}| \mu_{kt}), k=1,\ldots,K , t=1,\ldots,N, \\ g(\mu_{kt}) &= O_{kt} + \mathbf{x}_{kt}^\top{\boldsymbol{\beta}} + \zeta_{kt}. \end{split} \end{eqnarray*}

Here the spatio-temporal structure is modeled by $\zeta_{kt}$, and the first model we consider is similar to the main effect and interaction model proposed by Knorr-Held (2000) and is given by $\zeta_{kt}=\phi_k + \delta_t + \psi_{kt}$. Here, $\phi_k$ is a spatial main effect modeled with the CAR prior proposed by Leroux *and others* (2000) and given by (3.2), while $\delta_t$ is a temporal main effect also modeled by the CAR prior proposed by Leroux *and others* (2000). Finally, the space–time interaction terms are modeled by $\psi_{kt}\sim\mbox{N}(0, \sigma^2)$ and correspond to the type I interaction term suggested by Knorr-Held (2000). This model differs from the proposal of Knorr-Held (2000) in that Leroux rather than BYM (Besag *and others*, 1991) CAR priors are used for $(\phi_k, \delta_t)$, because this provides a closer comparison to model (3.1).

The second model we consider for case study 2 has area-specific linear temporal trends and is similar to that proposed by Bernardinelli *and others* (1995) and is given by $\zeta_{kt}=\phi_k + \delta_k t$. Here, $(\phi_k, \delta_k)$ are the intercept and slope for area $k$ and are both modeled by the Leroux CAR prior. Both models are fitted using MCMC simulation via the R package CARBayesST rather than INLA, because it allows a fairer comparison with our simulation based inference used for model (3.1).

## 4. Simulation study

We conduct a simulation study to quantify our model’s ability to correctly cluster areas based on their temporal trends, as well as comparing the performance of the (MC)$^3$ algorithm proposed here against a standard MCMC algorithm using Metropolis and Gibbs updating steps.

### 4.1. Data generation

**Fig. 1.**
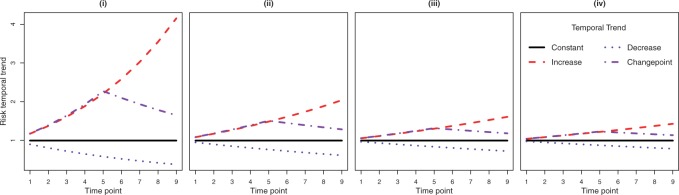
The true trends used in the four scenarios of the simulation study.

The study region is the $K=271$ IZ that make up the Greater Glasgow & Clyde health board (the study region for case study 2), and each simulated data set is generated on this region for $N=9$ time periods (as in case study 1). We consider the Poisson log-linear variant of model (3.1) in this study because it aligns with case study 2, and the exact model specification is presented in Appendix D of the supplementary material available at *Biostatistics* Online. In all simulations, we generate data with $S=4$ temporal trends: constant; linearly increasing; linearly decreasing; and a change point trend; which are the ones used in case study 1. We consider the four different simulation scenarios labeled (i) to (iv) in Figure 1, which have different levels of separations between the four trends. Scenario (i) corresponds to the biggest differences between the four trends and should be the easiest for correct clustering, where as in scenario (iv) the four trends are much more similar making clustering much harder. Within each of the four scenarios (i) to (iv) we consider three different allocation mechanisms of areas to trends via different inclusion probabilities ${\boldsymbol{\lambda}}=(\lambda_1, \lambda_2, \lambda_3, \lambda_4)$, where $\lambda_1$ is a constant, $\lambda_2$ is a linearly increasing, $\lambda_3$ is a linearly decreasing, and $\lambda_4$ is a change point trend. The three different allocation mechanisms are:

- (A) on average equal numbers of areas assigned to each trend—${\boldsymbol{\lambda}} = (0.25, 0.25, 0.25, 0.25)$;
- (B) all areas have a linearly decreasing trend—${\boldsymbol{\lambda}} = (0,0,1,0)$; and
- (C) on average unequal numbers of areas assigned to each trend—${\boldsymbol{\lambda}} = (0.5, 0.1, 0.2, 0.2)$.

Mechanism (B) allows us to assess model performance when a user includes trends in the model that are unnecessary. We generate 100 simulated data sets under each of the 12 scenario and allocation mechanism combinations, and fit model (3.1) with both the (MC)$^3$ and MCMC algorithms in each case. Inference is based on $1000$ samples, which are obtained by generating $60\,000$ samples and removing the first $50\,000$ as burn-in and thinning the remaining $10\,000$ by 10 to reduce the autocorrelation.

### 4.2. Main results of the simulation study

**Fig. 2.**
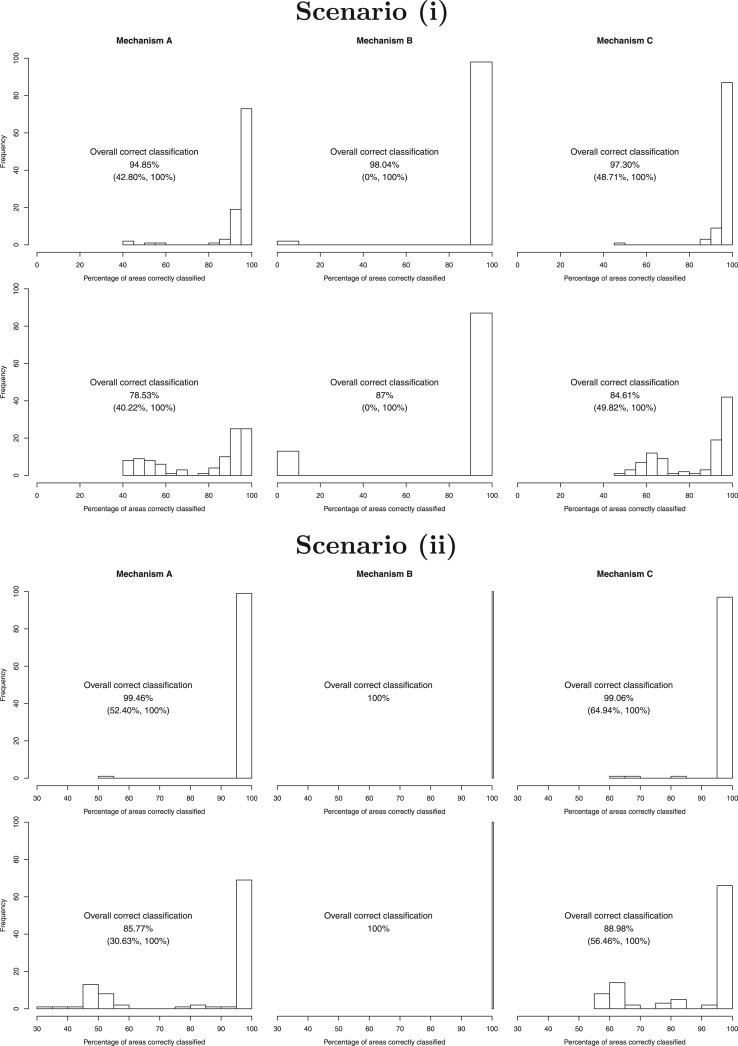
Histograms showing the percentage of areas allocated to the correct trend for each simulated data set in scenarios (i) (top) and (ii) (bottom). In each case, the top row relates to the (MC)$^{3}$ algorithm, and the bottom row relates to the standard MCMC algorithm. The numbers give the overall (all 100 data sets) correct classification percentages and the lowest and highest values for a single data set.

The main results of the simulation study are presented in Figures 2 (scenarios (i) and (ii)) and 3 (scenarios (iii) and (iv)), which display histograms of the correct classification percentages (i.e. the percentage of areas allocated to the correct trend function) across the 100 simulated data sets. For each scenario the left, middle, and right columns represent allocation mechanisms (A) to (C) respectively, while the results for the (MC)$^3$ algorithm are in the top row whilst those for the MCMC algorithm are in the bottom row. The numbers above each plot summarize the correct classification distributions, via the overall (all 100 data sets) correct classification percentages and the lowest and highest values for a single simulated data set.

**Fig. 3.**
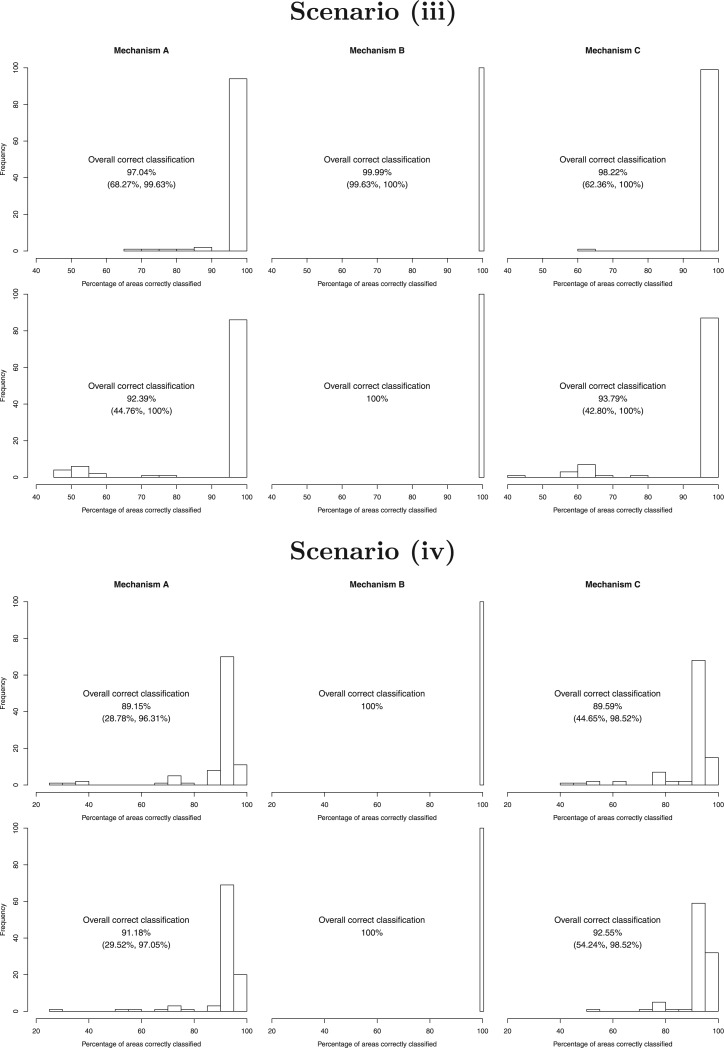
Histograms showing the percentage of areas allocated to the correct trend for each simulated data set in scenarios (iii) (top) and (iv) (bottom). In each case, the top row relates to the (MC)$^{3}$ algorithm, and the bottom row relates to the standard MCMC algorithm. The numbers give the overall (all 100 data sets) correct classification percentages and the lowest and highest values for a single data set.

Overall, allocation (B) proved to be the easiest to classify using both the (MC)$^3$ and MCMC algorithms, with almost 100% correct classification rates in all cases. This is likely to be because it only has one trend present in the data, making it easier to identify. For allocations (A) and (C) when more than one trend is present the (MC)$^3$ algorithm performs uniformly well, with overall classification probabilities ranging between 89.15% and 99.46% in all cases. In contrast, the MCMC algorithm generally performs poorer, with overall classification probabilities ranging between 78.53% and 93.79%. Furthermore, the (MC)$^3$ algorithm correctly classified over 80% of areas in an individual data set 95.5% of the time across all allocations (A) and (C), which compares to only 79.1% for the MCMC algorithm.

The MCMC algorithm exhibited poorest performance when the trends were most different (scenario (i)), which is likely to be because it got stuck in a local mode in the multimodal posterior distributions. Thus, its performance improved as the trends became more similar in scenarios (ii) and (iii), although by scenario (iv) the trends are very similar which makes the clustering harder. In contrast, the (MC)$^3$ algorithm exhibits its worst performance in scenario (iv) when the trends are most similar, which is to be expected. However, the (MC)$^3$ algorithm also performs slightly less well in scenario (i) compared with scenario (ii) despite the trends being more different in scenario (i), which again may suggest that for a small number of simulated data sets it struggles to move between the modes in the multimodal posterior distributions. This problem is far less pronounced however for the (MC)$^3$ algorithm compared with the MCMC algorithm, as the histograms of the correct classification probabilities in Figure 2 evidence.

### 4.3. Further results from the simulation study

The results above show that fitting model (3.1) with the (MC)$^{3}$ algorithm has excellent clustering ability across the range of scenarios considered, where as fitting with the MCMC algorithm gives less reliable performance. However, once the clustering of areas to temporal trends has been undertaken, interest lies in the magnitude and shape of the trends via the estimated $({\boldsymbol{\beta}}, {\boldsymbol{\gamma}})$ parameters. The accuracy of these parameter estimates, and hence the accuracy of the estimated trends, are summarized in Appendix D of the supplementary material available at *Biostatistics* Online and again shows that the (MC)$^{3}$ algorithm generally performs best. Finally, the simulation study has thus far fitted each model based on the assumption that all of the true trends observed in the data are included in model (3.1), which may not be realistic. Therefore Appendix D of the supplementary material available at *Biostatistics* Online presents the results from repeating the simulation study and fitting model (3.1) without the linearly decreasing trend, to see what happens when one of the true trends is omitted.

## 5. Results of the case studies

Inference for the clustering model is based on the (MC)$^{3}$ algorithm, because the simulation study showed it outperformed the simpler MCMC alternative. To enable a comparison of overall model fit, we apply the two competitor models outlined in Section 3.4, although we note that these models cannot undertake any clustering of the areas as our model can. These competitor models are fitted using MCMC simulation via the R package CARBayesST for consistency with the MCMC inferential approach utilized here, although INLAs could also be used.

### 5.1. Case study 1 results

The binomial logistic variant of model (3.1), $Y_{kt}\sim\mbox{Binomial}(N_{kt}, \theta_{kt})$, is used to model the number of children susceptible to measles $Y_{kt}$ in area $k$ and 2-year time period $t$, where $\theta_{kt}$ denotes the probability of being susceptible. Our questions of interest are: (i) did all IZs exhibit a change point in measles susceptibility as a result of the retraction of the Wakefield *and others* (1998) article in 2004; and (ii) did the change point occur in 2004 for all IZs, or was it earlier or later for some? To answer these questions we fit models with a change point trend (increasing then decreasing) as well as constant, linear increasing and linear decreasing trends, the latter three corresponding to no effect of the retraction of the Wakefield paper on measles susceptibility. To assess the sensitivity of the results to the choice of change point we fit three different models, where the change point is at: (A) 2002, (B) 2004, and (C) 2006. Additionally, to answer question (ii) we fit a fourth model (D) with two change point trends in 2004 and 2006. Inference for this study was based on 10 000 MCMC samples, which were generated by burning in each chain for 200 000 samples and then thining the next 100 000 samples by 10 to reduce their autocorrelation.

The overall fit of each model is summarized by the Watanabe-Akaike Information Criteron (WAIC, Watanabe, 2010), which together with the effective number of independent parameters (in brackets) are given by: (A) 53 780 (801), (B) 52 708 (490), (C) 53 481 (757), and (D) 52 720 (497). Model (B) fits the data best, which agrees with our prior knowledge that the retraction of the Wakefield paper occurred in 2004. Additionally, model (D) which includes a second change point to model (B) does not reduce the WAIC further, suggesting that having the 2004 change point alone best fits the data (none of the IZs were assigned to the 2006 change point trend in Model (D)). For a comparison of overall model fit, the model similar to that proposed by Knorr-Held (2000) and outlined in Section 3.4 has a WAIC of 52 554 (420), which suggests that it fits the data slightly better than the mixture model proposed here. However, it does not allow the areas to be clustered based on shared temporal trends.

For model (B), the best fitting clustering model, 1233 of the 1235 IZs are allocated to the trend with a change point in 2004, whilst the remaining two IZs have a constant trend. The posterior probabilities that those areas are allocated to the 2004 change point range between 0.34 and 1, with a median posterior classification probability of 0.99. In contrast, the two areas assigned to the constant trend were done so with probabilities 0.34 and 0.36, respectively, so there is large uncertainty in their classifications. The allocations of areas to trends for models (A) to (C) are similar and are presented in Appendix E of the supplementary material available at *Biostatistics* Online.

**Fig. 4.**
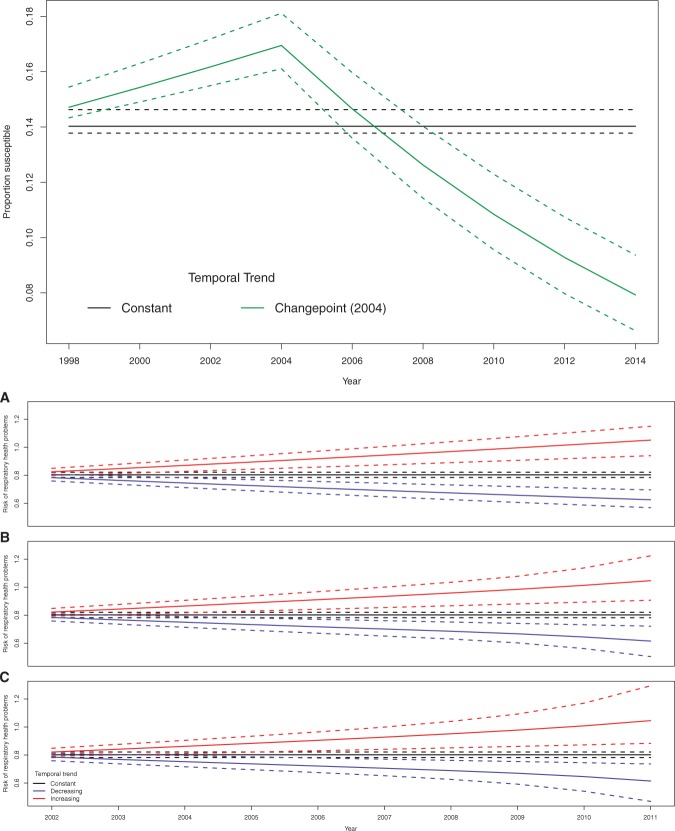
The estimated temporal trends and 95% credible intervals (dotted lines) from the measles susceptibility (top) and respiratory hospitalization (bottom) case studies.

The estimated trend functions in model (B) are displayed in the top panel of Figure 4, where the increasing and decreasing trends are not shown as no IZs were allocated to them. The trends are plotted on the scale of the proportion of pre-school children susceptible to measles, that is $\hat{\theta}_t = \frac{\exp\left(\hat{\beta}_1+ \hat{f}_s(t|\hat{{\boldsymbol{\gamma}}}_s)\right)}{1 + \exp\left(\hat{\beta}_1 + \hat{f}_s(t|\hat{{\boldsymbol{\gamma}}}_s)\right)}$. The figure shows an increase in susceptibility from 0.147 to 0.169 between 1998 and 2004 before a decrease in subsequent years, with susceptibility at its lowest (0.079) in 2014. These results suggest that almost all areas were affected by the retraction of the Wakefield article in 2004, as only two IZs were not classified to the 2004 change point trend. As a result, it suggests that in this case fitting a single change point trend model for all areas would likely fit the data nearly as well as our clustering model.

### 5.2. Case study 2 results

The Poisson log-linear variant of model (3.1), $Y_{kt}\sim\mbox{Poisson}(E_{kt}\theta_{kt})$, is used to model the number of respiratory hospital admissions $Y_{kt}$ in IZ $k$ and year $t$, where $\theta_{kt}$ is the risk of hospitalization relative to $E_{kt}$. Our aim is to determine which areas in Greater Glasgow have exhibited an increased risk of disease, which have exhibited a decreased risk, and which have showed no change over the 10-year study period, as well as estimating what impact these changes have had on health inequalities. Therefore we fit $S=3$ trends in this model, a monotonic increase, a monotonic decrease, and no change. To assess the sensitivity of the results, we fit three separate models: (A) linear trends, (B) monotonic trends with 1 internal knot, and (C) monotonic trends with two internal knots; with only 1 or 2 evenly spaced knots considered due to a small number of time points. Inference for this study was based on 10 000 MCMC samples, which were generated by burning in each chain for 100 000 samples and then thining the next 100 000 samples by 10 to reduce their autocorrelation.

The WAIC and the effective number of independent parameters (in brackets) for the three models are: (A) 21 649 (829), (B) 21 625 (818), and (C) 21 620 (814), suggesting that all three models exhibit similar fits to the data. For comparison, the main effect and interaction model outlined in Section 3.4 has a WAIC of 20 349 (1006), while the area specific linear trends model has a WAIC of 21 126 (634). Thus both these models fit the data slightly better than the mixture model proposed here, but do not allow any clustering of areas based on shared temporal trends.

The bottom panel of Figure 4 displays the estimated temporal trends and 95% credible intervals on the risk scale, namely $\hat{\theta}_t = \exp\left(\hat{\beta}_1 +\hat{f}_s(t|\hat{{\boldsymbol{\gamma}}}_s)\right)$, where the three components of the figure show estimates from the linear (A) and monotonic ((B) and (C)) trend models. Models (B) and (C) show almost no curvature and linear lines fit easily within the 95% credible intervals, which re-enforces the similarity in their model fits observed above. The allocation of the 271 IZs to the 3 temporal trends shows little sensitivity between the three models, with pairwise agreement ranging between 96.3% and 98.5% (details are given in Appendix E of the supplementary material available at *Biostatistics* Online). Just under half of the areas are allocated to the constant trend suggesting no change in the risk of respiratory hospitalization over time, with the remaining areas almost equally assigned between the increasing and decreasing risk trends. These equal numbers reflect the raw data plot presented in Appendix A of the supplementary material available at *Biostatistics* Online, which shows similar levels of spatial variation in the left and right ends of the plot. The spatial standard deviations in the estimated risk $\hat{\theta}_{kt}$ are 0.30 in both 2002 and 2011, which suggest that the magnitude of the health inequalities are unchanged over the 10-year period.

**Fig. 5.**
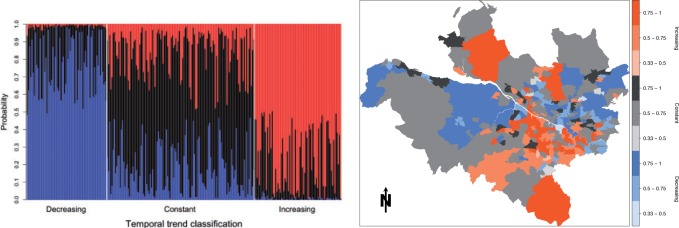
The left panel shows the posterior probabilities that each area (given on $x$-axis) is assigned to each trend, while the right panel shows the classification based on the maximum *a posteriori* probabilities. For the latter, the darker the shading the higher the posterior probability for that trend. All results are from the model with a spline trend with $q=2$ knots.

Finally, the spatial classification of areas to the three trends and the posterior classification probabilities are displayed in Figure 5, where the results relate to the spline trend with $q=2$ internal knots. The left panel shows the posterior probability that each area is assigned to each trend, with the three parts of that figure grouping areas according to their maximum *a posteriori* trend. The figure shows there is relatively little posterior uncertainty in the classifications for areas assigned to the increasing and the decreasing trends, with classification probabilities between 0.5 and 1 and the remaining posterior probability share being mainly taken by the constant trend. In contrast, the classifications of areas to the constant trend are much more uncertain, with probabilities mostly lying between 0.5 and 0.75, with the remaining probability shared equally between the increasing and decreasing trends. This is likely because the constant trend is the middle of the three possibilities, hence the greater uncertainty. The map in the right panel of Figure 5 shows the spatial classification, where the darker the shading the higher the posterior classification probability. For example, the darkest shading indicates that the posterior probability for that trend was above 0.75. Interestingly, the map shows spatial grouping of the trends, with areas north-east of the river Clyde typically showing an improvement in terms of risk while areas south-east of the river predominantly exhibit increased levels of risk. This spatial clustering was not enforced by the model, but one also observes that many pairs of neighboring areas that exhibit different trends (e.g. increasing and decreasing), suggesting that enforcing spatial clustering would not have been appropriate here.

## 6. Discussion

We have presented a novel spatio-temporal mixture model for clustering areas based upon shared parametric or shape-constrained temporal trends, which allows specific hypotheses to be tested about the data under study. As the main goal of our model was to identify groups of areas that exhibit similar temporal trends, our approach is complementary and not in competition with other space–time models such as Knorr-Held (2000) and Bernardinelli *and others* (1995), whose primary aims are spatio-temporal risk estimation rather than trend-based clustering. Existing approaches for trend-based clustering such as Choi *and others* (2011) have been based on unconstrained forms such as random walks, but this could result in two or more of the estimated trends having similar shapes, reducing the utility of the clustering. We used the maximum *a posteriori* rule for choosing which of the $S$ trends an area is allocated to because it produces a hard classification, and the simulation study in Section 4 showed this performs well across a range of scenarios. This study also showed the utility of the (MC)$^3$ model fitting algorithm we have developed, which consistently outperformed a simpler MCMC alternative. The model, together with the data for case study 2, are available at https://github.com/GNapier/SpaceTimeClusteringDiseaseTrends to make this research reproducible.

The key insight from the measles susceptibility case study is that there was a consistent Scotland-wide effect of the retraction of the Wakefield *and others* (1998) paper in 2004, with all but two of the IZ being assigned to the change point trend. Between the articles publication in 1998 and its retraction in 2004 this trend showed around a 2.2% increase in measles susceptibility, rising from 14.8% in 1998 to a peak of 17% in 2004. Following the retraction of the article the measles susceptibility rate has continued to fall, being at an all-time low in 2014 of 7.9%. Thus it seems that while there is spatial variation in the rates of measles susceptibility, the temporal trends are very consistent showing little between IZ variation across Scotland.

The key insight from the respiratory hospitalizations case study is that the level of health inequality across the Greater Glasgow & Clyde health board has hardly changed from 2002 to 2011, with estimated spatial standard deviations in risk of 0.30 in both 2002 and 2011. Furthermore, just under half of the IZ have shown no change in the risk of admission over the 10-year time period, with the remaining 50% of areas being almost equally divided into increasing and decreasing trends. However, most of the areas that exhibited an increased risk are in the south of Glasgow, where as most of the decreased risks are in the north of the city. This city-wide north–south divide is an interesting finding, and the National Health Service Scotland will be interested in understanding the reasons for this phenomenon.

The methodological framework outlined here has allocated each spatial unit independently to a temporal trend, because this does not force areas close together to exhibit similar trends. However if one had such *a priori* spatial clustering beliefs, then the model could be expanded to take account of this, perhaps by extending the *a priori* clustering probabilities ${\boldsymbol{\lambda}}$ to ${\boldsymbol{\lambda}}_k$ and forcing them to be correlated spatially. The other key area of future development surrounds the overall quantification of health inequalities, and the work here has examined these in the context of a single health outcome. However, an in-depth study of health inequalities requires the consideration of multiple diseases simultaneously, and thus we will extend the methodology developed here to the multivariate domain. Within the United Kingdom the biggest killers are cancer and cerebrovascular coronary heart and respiratory diseases (http://www.gov.scot/Topics/Statistics/Browse/Health/TrendMortalityRates), and thus a multivariate extension of the methodology will focus on overall health inequalities in these four diseases. Finally, a computational development would be to improve the computational efficiency of the (MC)$^{3}$ algorithm by making use of multiple CPU cores.

## Supplementary Material

kxy024_Supplementary_Data
