# Supplementary material for: A Bayesian space–time model for clustering areal units based on their disease trends
Source: Biostatistics. 2018 Jun 18;20(4):681–97. doi: 10.1093/biostatistics/kxy024 (PMC6797054; doi:10.1093/biostatistics/kxy024)
Supplement: kxy024_Supplementary_Data [file kxy024_supplementary_data.pdf]

Biostatistics (2018), **0**, 0, *pp.* 1–17
doi:10.1093/biostatistics/BioStatsSupplementaryMaterialsRevisionDL

A Bayesian space-time model for clustering areal units based on their disease trends Supplementary Materials

GARY NAPIER*, DUNCAN LEE

School of Mathematics and Statistics, University of Glasgow, G12 8SQ, UK

Gary.Napier@glasgow.ac.uk

CHRIS ROBERTSON

Department of Mathematics and Statistics, University of Strathclyde, G1 1XH, UK

ANDREW LAWSON

Department of Public Health Sciences, Medical University of South Carolina, USA

Introduction

This supplementary material has the following sections. Appendix A presents additional data description for the two case studies, while Appendix B describes the $(\mathrm{MC})^3$ estimation algorithm used to fit the model. Appendix C summarises the computational demand of fitting our model to data of different sizes, while Appendix D presents the data generation and additional results from the simulation study. Appendix E presents additional sensitivity analyses for the two case studies.

*To whom correspondence should be addressed.

© The Author 2018. Published by Oxford University Press. All rights reserved. For permissions, please e-mail: journals.permissions@oup.com

Appendix A - Additional data description for the two case studies

A.1 Measles susceptibility case study

The study region is Scotland, and a map with the main towns and cities is presented in Figure 1. Scotland has a population of 5.4 million people, and for this study has been partitioned into $K = 1235$ non-overlapping intermediate zones (IZ), which are a key geography designed by the Scottish Government for distributing small-area statistics. The spatial pattern in the proportion susceptible (un-vaccinated) $\{\hat{\theta}_{kt}\}$ in 2004 is displayed in Figure 2, which shows that the less densely populated regions in the north and west of Scotland appear to have higher susceptibility rates compared to the more densely populated regions, such as Glasgow and Edinburgh. One of the reasons for this could be that general practitioners (doctors) and health centres are not as accessible in these remote rural regions, and so in combination with the negative press towards the MMR vaccine, parents in those areas may have been less likely to have their children vaccinated. The temporal trend in $\{\hat{\theta}_{kt}\}$ for all areas is displayed in panel (a) of Figure 3, which shows a clear change point in 2004 which corresponds to the year of the retraction of the discredited article by Wakefield.

A.2 Respiratory hospitalisation case study

The study region for the respiratory hospitalisation case study is the Greater Glasgow and Clyde Health Board, which is displayed as the shaded region in Figure 4. The region has been partitioned into $K = 271$ non-overlapping intermediate zones, and the spatial pattern in the SMR, $\hat{\theta}_{kt}$, in 2005 is displayed in Figure 5. The spatial pattern shows elevated SMR values in the east of the city and just south of the river Clyde (the white line running south-east), which are some of the poorest parts of the study region. The temporal trends are displayed in panel (b) of Figure 3, and show no clear trends over the 10 year period.

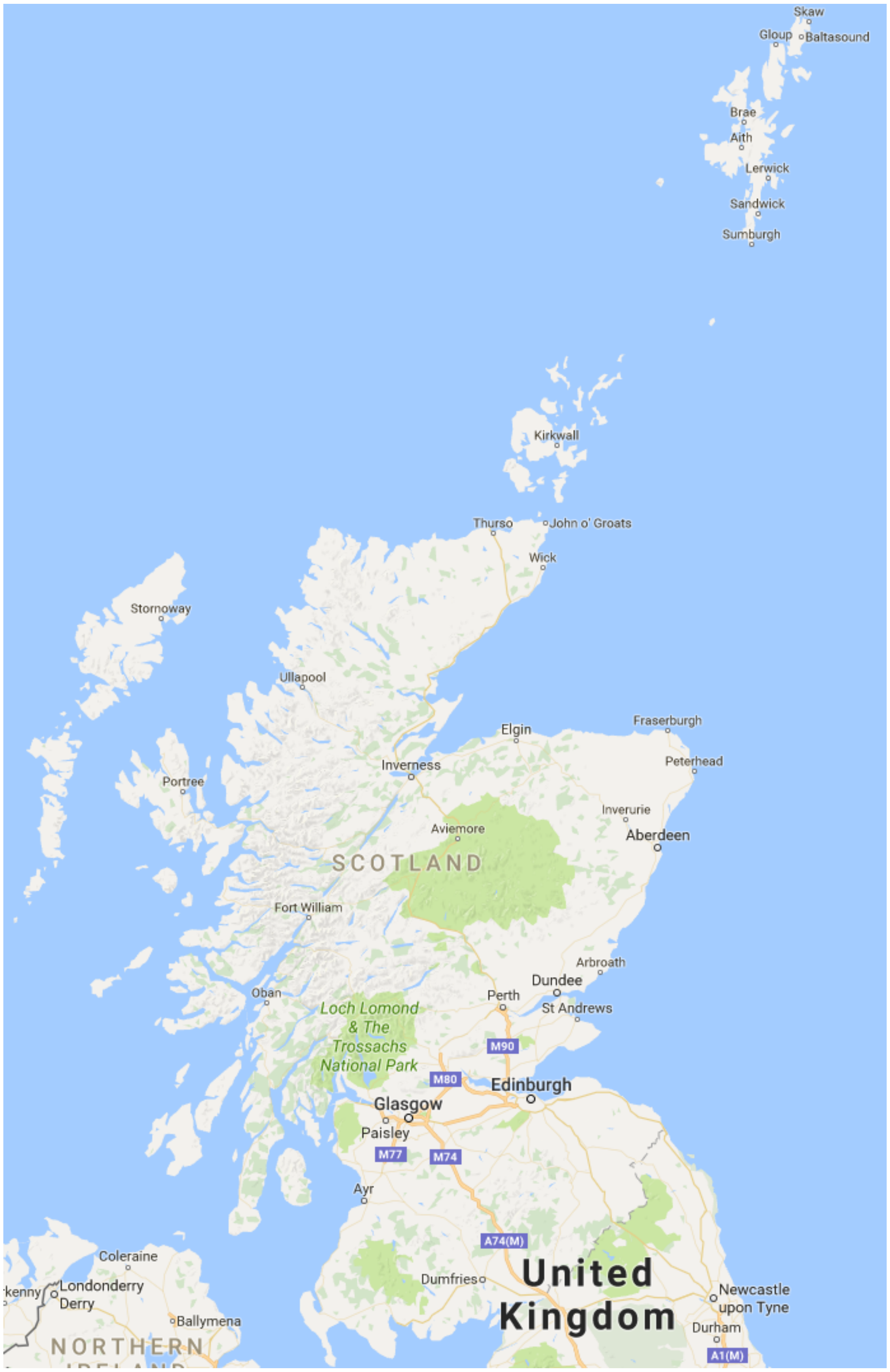

Fig. 1: Map of Scotland showing the main towns and cities.

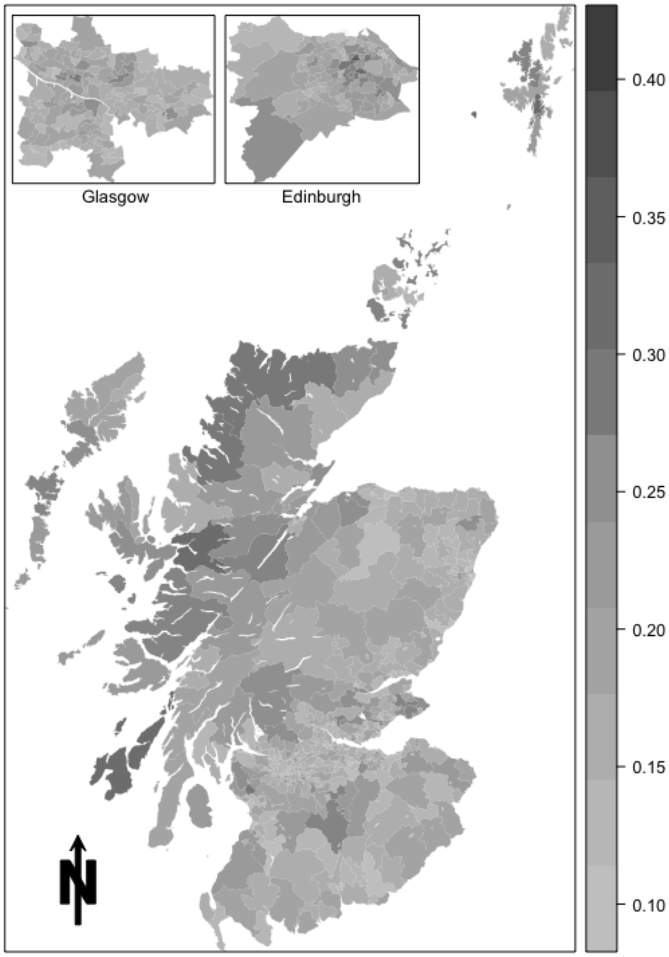

Fig. 2: Spatial pattern in the estimated proportions $\hat{\theta}_{kt}$ of pre-school children susceptible to measles in Scotland in 2004. The panels in the top-left corner highlight the cities Glasgow and Edinburgh in the centre of Scotland.

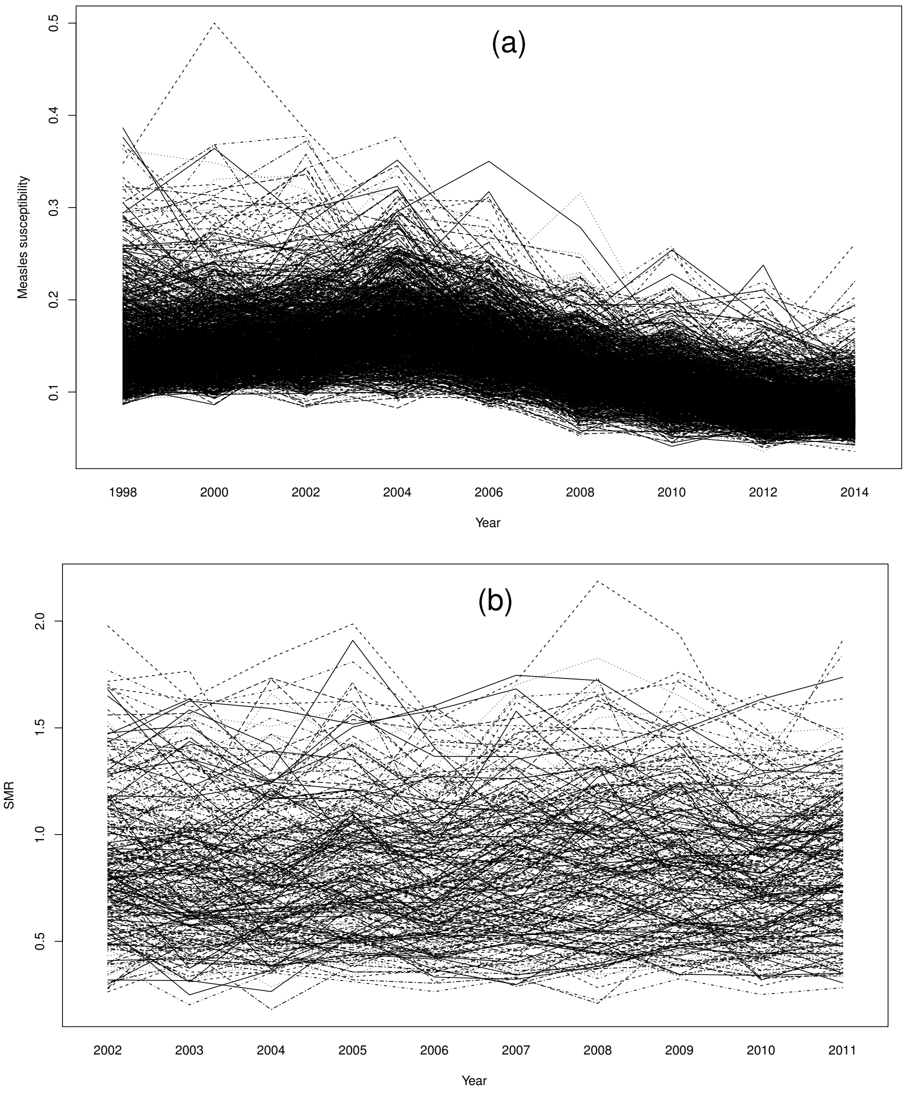

Fig. 3: Temporal trends in: (a) susceptibility to measles $\hat{\theta}_{kt}$; and (b) risk of hospitalisation due to respiratory disease.

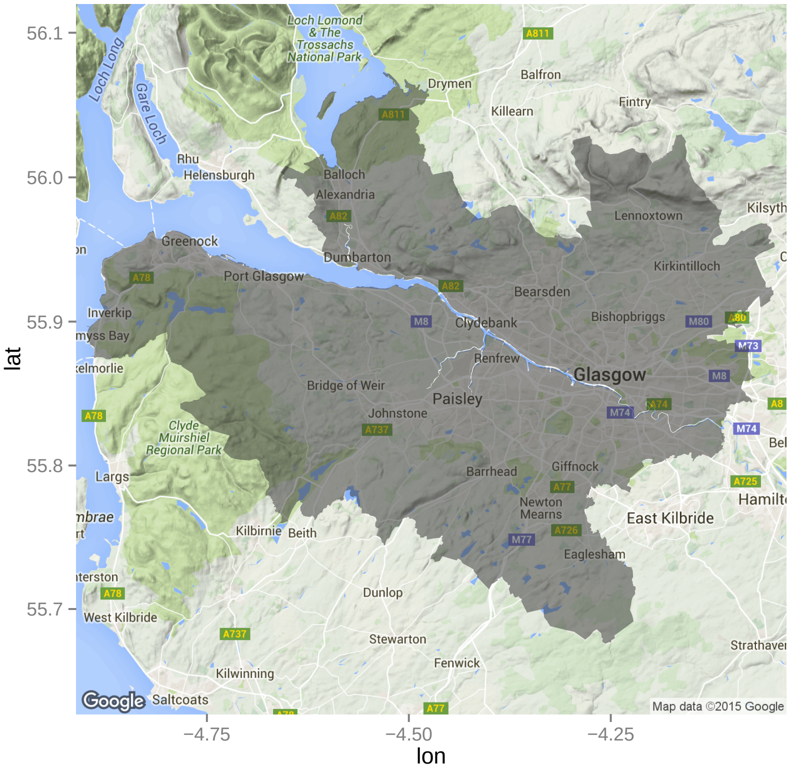

Fig. 4: The Study region of the Greater Glasgow and Clyde Health Board (shaded region) overlaid on a Google map.

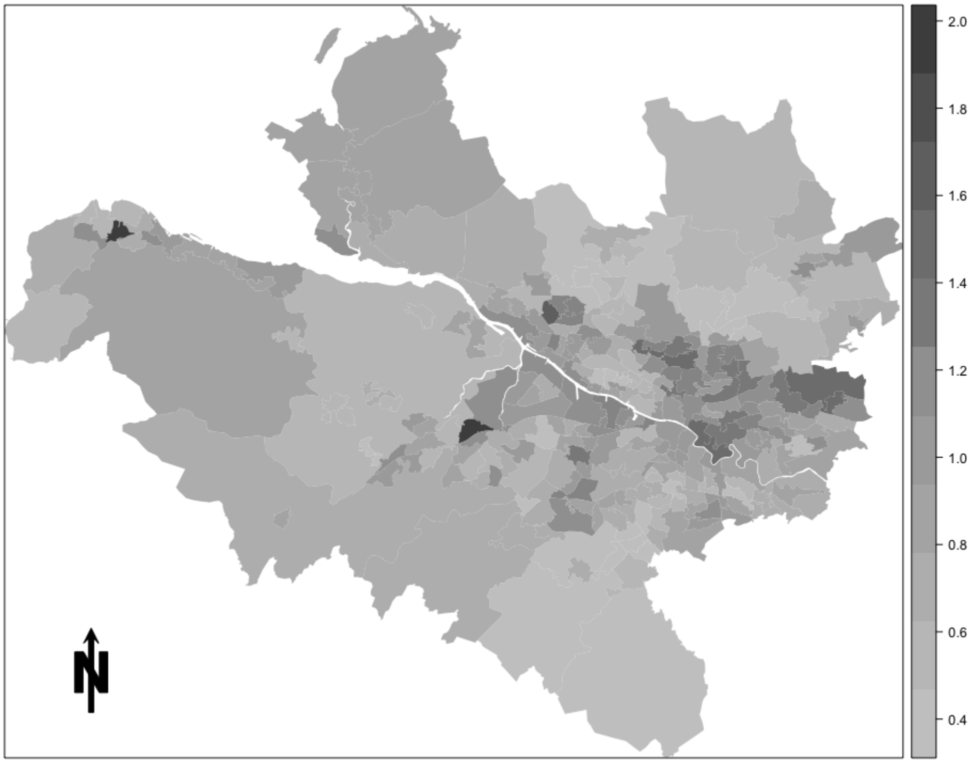

Fig. 5: Spatial pattern in the SMR $\{\hat{\theta}_{kt}\}$ for respiratory hospitalisation in 2005.

Appendix B - (MC)^3 estimation algorithm

For our (MC)^3 algorithm M Markov chains $\mathcal{C} = (\mathcal{C}_1, \ldots, \mathcal{C}_M)$ are run in parallel with temperatures $T_1 = 1 > T_2 > \ldots > T_M > 0$. The first chain with $T_1 = 1$ is known as the cold chain, and is the one upon which final posterior inference is based. Denote the collection of model parameters for the i-th iteration of the m-th chain as $\Psi_{\mathcal{C}_{mi}} = \{\boldsymbol{\beta}_{\mathcal{C}_{mi}}, \boldsymbol{\phi}_{\mathcal{C}_{mi}}, \boldsymbol{\omega}_{\mathcal{C}_{mi}}, \boldsymbol{\lambda}_{\mathcal{C}_{mi}}, \rho_{\mathcal{C}_{mi}}, \tau^2_{\mathcal{C}_{mi}}, \boldsymbol{\gamma}_{\mathcal{C}_{mi}}\}$, where $\boldsymbol{\gamma}_{\mathcal{C}_{mi}}$ is the complete vector of parameters comprising the S trend functions. Then the (MC)^3 algorithm is defined as follows.

(MC)^3 Algorithm

1. Specify initial values, $\Psi_{\mathcal{C}_{m0}}$ for each parameter in each Markov chain for $m = 1, \ldots, M$.

2. Repeat the following steps for $i = 1, \ldots, n$ iterations of the sampling scheme.

   (a) At iteration i repeat the following steps for each Markov chain for $m = 1, \ldots, M$, and each model parameter $\psi_{\mathcal{C}_m} \in \Psi_{\mathcal{C}_m}$.

   i. Sample a proposal value $\tilde{\psi}_{\mathcal{C}_{mi}}$ from a proposal distribution $q(\tilde{\psi}_{\mathcal{C}_{mi}} | \psi_{\mathcal{C}_{mi}})$.

   ii. Accept $\tilde{\psi}_{\mathcal{C}_{mi}}$ as the next value in the chain, that is set $\psi_{\mathcal{C}_{mi+1}} = \tilde{\psi}_{\mathcal{C}_{mi}}$ with probability $\min(1, \alpha_1)$, where

$$\alpha_1 = \frac{p(\tilde{\psi}_{\mathcal{C}_{mi}})^{T_m} q(\psi_{\mathcal{C}_{mi}} | \tilde{\psi}_{\mathcal{C}_{mi}})}{p(\psi_{\mathcal{C}_{mi}})^{T_m} q(\tilde{\psi}_{\mathcal{C}_{mi}} | \psi_{\mathcal{C}_{mi}})},$$

   and $p(\cdot)$ denotes the full conditional distribution of $\tilde{\psi}_{\mathcal{C}_{mi}}$ or $\psi_{\mathcal{C}_{mi}}$.

   (b) To couple the chains randomly select two of the chains, say $\tilde{\Psi}_{\mathcal{C}_{ji}}$ and $\tilde{\Psi}_{\mathcal{C}_{ki}}$.

   i. The proposal values for a Metropolis type move are $\tilde{\Psi}_{\mathcal{C}_{ji}} = \Psi_{\mathcal{C}_{ki}}$ and $\tilde{\Psi}_{\mathcal{C}_{ki}} = \Psi_{\mathcal{C}_{ji}}$.

   ii. Swap the chains with probability $\min(1, \alpha_2)$, where

$$\alpha_2 = \frac{p(\tilde{\Psi}_{\mathcal{C}_{ki}})^{T_k} p(\tilde{\Psi}_{\mathcal{C}_{ji}})^{T_j}}{p(\Psi_{\mathcal{C}_{ki}})^{T_k} p(\Psi_{\mathcal{C}_{ji}})^{T_j}}.$$

Note that for parameters that can be Gibbs sampled the temperatures do not apply, and the acceptance rate is always equal to one. From the sampling scheme it is clear that increasing the temperature level T_m increases the acceptance probability α_1. The acceptance rates for accepting a new proposed value, $\tilde{\psi}_{\mathcal{C}_{mi}}$, are controlled by updating the standard deviation of the proposal distribution $q(\cdot)$, such that an acceptance rate of 40-50% is achieved. Through simulation testing we found that swapping chains with an acceptance rate of 20-30% was sufficient to remove the multimodality issue, which is similar to the results of Atchadé *and others* (2011). In order to obtain such an acceptance rate geometric spacing of the temperatures was used, that is $T_{m+1} = c \cdot T_m$, where $T_1 = 1$ and $0 < c < 1$. The constant c is updated during the algorithm to ensure the spacing between the temperatures of the chains provides an acceptance rate of 20-30%, such that, if the acceptance rate is greater than 30%, the difference between the temperatures will widen, and hence shrink if the acceptance rate of swapping chains is less than 20%. Choosing the number of chains M is an extra choice to be made in this algorithm and can be application specific (Altekar *and others*, 2004), and here $M = 4$ appeared to work well following exploratory runs of the algorithm for both the simulated and real data sets. Tuning of the proposal distributions to have the acceptance rates discussed above is automatically undertaken in the algorithm, meaning the user does not need to manually tune the algorithm.

Appendix C - Computation time and scalability

Table 1 displays the computation times for fitting the mixture model proposed in the main paper to data of various sizes, which illustrates the scalability of our approach. Run times are provided for both the MCMC and the $(\text{MC})^3$ algorithms, and in all cases the models are run with $S = 4$ temporal trends on regular spatio-temporal grids. Inference in all cases was based on $100,000$ iterations, and the $(\text{MC})^3$ algorithm had $M = 4$ parallel chains (as used in the simulation and real data studies) resulting in $400,000$ iterations in total. The timings were carried out using

an Apple iMac computer with a 3.2 GHz Intel Core i5 processor and 16 GB MHz DDR3 memory. The table displays run times for data sizes between 1,000 (10×10 spatial grid with 10 time periods) and 50,000 (50×50 spatial grid with 20 time periods) data points, and the maximum run time for 100,000 iterations is under 18 hours. The Table shows that run times increase with increasing numbers of data points as expected, and that the $(\mathrm{MC})^3$ algorithm is around 3 times slower than the MCMC algorithm despite generating 4 times as many iterations (it is based on 4 parallel chains).

Table 1: Summary of the time taken to run the mixture model in seconds (in hours, minutes and second in brackets) on a regular grid with different square grid sizes (K), number of time periods (N) and total number of data points (N.all).

K	N	N.all	Timing in seconds (h:m.s) $(\mathrm{MC})^3$	MCMC
$K = 10 \times 10 = 100$	$N = 10$	$1,000$	1,523 (25.23)	492 (8.12)
$K = 10 \times 10 = 100$	$N = 20$	$2,000$	2,508 (41.48)	803 (13.23)
$K = 20 \times 20 = 400$	$N = 10$	$4,000$	5,003 (1:23.23)	1,637 (27.17)
$K = 20 \times 20 = 400$	$N = 20$	$8,000$	10,003 (2:46.43)	3,063 (51.3)
$K = 30 \times 30 = 900$	$N = 20$	$18,000$	22,396 (6:13.16)	6,908 (1:55.8)
$K = 30 \times 30 = 900$	$N = 30$	$27,000$	33,730 (9:22.10)	10,065 (2:47.45)
$K = 40 \times 40 = 1600$	$N = 30$	$48,000$	62,270 (17:17.50)	17,562 (4:52.42)
$K = 50 \times 50 = 2500$	$N = 20$	$50,000$	64,010 (17:46.50)	18,648 (5:10.48)

Appendix D - Data generation and additional results for the simulation study

D.1 - Data generation

The data generated in the simulation study come from the following Poisson log-linear variant of the general model (3.1) in the main paper.

$$Y_{kt} \sim \text{Poisson}(E_{kt}R_{kt}), \quad k = 1, \ldots, K = 271, \quad t = 1, \ldots, N = 9,$$

$$\ln(R_{kt}) = \beta_0 + \phi_k + \sum_{s=1}^{4} \omega_{ks} f_s(t|\boldsymbol{\gamma}_s),$$

where $E_{kt} = 100 \ \forall \ k$ and t. The random effects $\boldsymbol{\phi}$ are generated from the Leroux CAR model outlined by (3.2) in the main paper, with $\rho = 0.99$ and $\tau^2 = 0.001$. The temporal trend functions

$f_s(t|\boldsymbol{\gamma}_s)$ used to generate the data are presented in Figure 2 in the main paper, and show constant, linearly increasing, linearly decreasing and change point forms.

D.2 - Accuracy of the trend estimation

The trends $f_s(t|\boldsymbol{\gamma}_S)$ estimated in the simulation study are represented by:

1. Constant trend - β_1.

2. Linear increasing trend - $\beta_1 + \gamma_1 t$, with $\gamma_1 > 0$.

3. Linear decreasing trend - $\beta_1 + \gamma_2 t$, with $\gamma_2 < 0$.

4. Change point trend - $\beta_1 + \gamma_3 t + \gamma_4 (t-5)_+$, with $\gamma_3 > 0$ and $\gamma_4 < 0$.

Tables 2 (scenarios (i) and (ii)) and 3 (scenarios (iii) and (iv)) display the bias, root mean square error (RMSE), and coverage probabilities of the 95% credible intervals for the parameters $(\beta_1, \gamma_1, \ldots, \gamma_4)$ associated with the 4 temporal trends, from using both the $(\text{MC})^3$ and MCMC algorithms. Note, that in allocation mechanism (B) the only trend present in the data is the linearly decreasing one, and hence $(\gamma_1, \gamma_3, \gamma_4)$ do not have true values for the computation of the metrics described above. Unsurprisingly, the results mirror those observed in the classification percentages presented in the main paper, and have the following general trends.

For allocation mechanism (B) where all areas are assigned to a decreasing trend the $(\text{MC})^3$ algorithm performs well in all scenarios, with all 95% credible intervals exhibiting close to the nominal coverage level and all estimates having negligible biases. Similar results are observed for the MCMC algorithm, with the exception being scenario (i) where the covarage probabilities drop to around 80% and the RMSE values are larger compared with the $(\text{MC})^3$ algorithm. For allocation mechanisms (A) and (C) the $(\text{MC})^3$ algorithm consistently outperforms the MCMC algorithm in scenarios (i) to (iii), with lower RMSE values in 29 out of 30 cases. This trend is

reversed in scenario (iv) where the MCMC algorithm gives the lowest RMSE values in 9 out of 10 cases, although in this case the magnitudes in the differences are generally not large. Finally, the $(MC)^3$ algorithm has coverage probabilities close to the nominal 95% levels in most cases, where as for the MCMC algorithm this is only true for scenario (iv). For scenarios (i) to (iii) the MCMC algorithm produces coverage probabilities between 11% and 89%, which is due to the relatively poor parameter estimation as measured by RMSE.

Table 2: Bias, root mean square error (RMSE) and coverage probabilities of the 95% credible intervals from the parameters of the temporal trends and the intercept term for scenarios (i) and (ii) and allocation mechanisms (A) to (C) using the $(MC)^3$ (left) and MCMC (right) algorithms.

**Scenario**	**Parameter**	**$(MC)^3$ algorithm**			**MCMC algorithm**		
		Bias	RMSE	Coverage	Bias	RMSE	Coverage
**(i) A**	β_1	0.0041	0.0192	83%	0.0378	0.0584	30%
	γ_1	0.0084	0.0131	53%	0.0198	0.0226	11%
	γ_2	-0.0045	0.0221	95%	-0.0305	0.0536	65%
	γ_3	-0.0051	0.0087	83%	-0.0082	0.0133	55%
	γ_4	0.0170	0.0523	85%	0.0853	0.1428	52%
**(i) B**	β_1	-0.0075	0.0506	92%	-0.0441	0.1222	80%
	γ_2	0.0011	0.0114	92%	0.0139	0.0387	79%
**(i) C**	β_1	0.0035	0.0130	84%	0.0280	0.0450	38%
	γ_1	0.0098	0.0151	54%	0.0197	0.0244	27%
	γ_2	-0.0014	0.0124	99%	-0.0314	0.0553	63%
	γ_3	-0.0034	0.0077	88%	-0.0096	0.0147	49%
	γ_4	0.0079	0.0300	86%	0.0839	0.1390	48%
**(ii) A**	β_1	0.0004	0.0052	96%	0.0131	0.0244	68%
	γ_1	-0.0002	0.0013	97%	-0.0013	0.0025	70%
	γ_2	-0.0005	0.0047	98%	-0.0124	0.0242	72%
	γ_3	-0.0005	0.0022	99%	-0.0032	0.0067	73%
	γ_4	0.0031	0.0176	95%	0.0341	0.0658	71%
**(ii) B**	β_1	-0.0001	0.0047	95%	0.0003	0.0047	94%
	γ_2	-0.0001	0.0009	96%	-0.0001	0.0009	94%
**(ii) C**	β_1	-0.0002	0.0028	98%	0.0082	0.0175	68%
	γ_1	-0.0002	0.0015	99%	-0.0009	0.0022	89%
	γ_2	0.0000	0.0010	99%	-0.0147	0.0276	65%
	γ_3	-0.0002	0.0026	98%	-0.0025	0.0061	72%
	γ_4	0.0031	0.0230	99%	0.0337	0.0653	66%

D.3 - Results from not including a true trend in the model

The simulation study presented above has fitted model (3.1) with all temporal trends that generated the data included as possible trends in the model. However, this does not illustrate

Table 3: Bias, root mean square error (RMSE) and coverage probabilities of the 95% credible intervals from the parameters of the temporal trends and the intercept term for scenarios (iii) and (iv) and allocation mechanisms (A) to (C) using the $(MC)^3$ (left) and MCMC (right) algorithms.

**Scenario**	**Parameter**	**$(MC)^3$ algorithm** Bias	RMSE	Coverage	**Standard algorithm** Bias	RMSE	Coverage
**(iii) A**	β_1	0.0002	0.0044	100%	0.0024	0.0084	86%
	γ_1	0.0000	0.0011	100%	-0.0003	0.0013	94%
	γ_2	-0.0007	0.0031	100%	-0.0038	0.0110	87%
	γ_3	-0.0006	0.0021	100%	-0.0007	0.0026	90%
	γ_4	0.0033	0.0148	99%	0.0092	0.0276	88%
**(iii) B**	β_1	-0.0004	0.0050	96%	0.0001	0.0050	93%
	γ_2	0.0000	0.0009	95%	-0.0001	0.0008	93%
**(iii) C**	β_1	-0.0001	0.0037	99%	0.0017	0.0068	86%
	γ_1	0.0001	0.0016	99%	-0.0002	0.0015	97%
	γ_2	-0.0005	0.0030	100%	-0.0037	0.0106	86%
	γ_3	-0.0001	0.0020	98%	-0.0002	0.0024	92%
	γ_4	0.0001	0.0095	99%	0.0085	0.0286	83%
**(iv) A**	β_1	-0.0003	0.0043	100%	-0.0008	0.0037	99%
	γ_1	0.0005	0.0015	98%	0.0002	0.0012	97%
	γ_2	-0.0015	0.0060	99%	-0.0011	0.0059	98%
	γ_3	-0.0010	0.0026	100%	0.0003	0.0019	99%
	γ_4	0.0022	0.0085	100%	-0.0002	0.0085	98%
**(iv) B**	β_1	-0.0008	0.0050	96%	0.0000	0.0046	94%
	γ_2	-0.0001	0.0009	96%	-0.0001	0.0008	94%
**(iv) C**	β_1	0.0003	0.0040	100%	-0.0005	0.0038	96%
	γ_1	0.0009	0.0030	99%	-0.0001	0.0016	98%
	γ_2	-0.0010	0.0036	99%	-0.0019	0.0077	97%
	γ_3	-0.0017	0.0032	100%	0.0003	0.0022	99%
	γ_4	0.0067	0.0157	98%	0.0007	0.0100	96%

what happens if an important trend that is in the data is excluded in the model fitting. Therefore we repeated the simulation study by fitting model (3.1) without the linearly decreasing trend using the $(MC)^3$ estimation algorithm.

The results are displayed in Table 4 in two parts; (a) the percentages of those areas that were generated as having a decreasing trend (not included in the model) that were classified to the trends included in the model; and (b) for areas whose true trend was included in the model the percentage correct classification. The results from part (a) suggest that for all scenarios and allocation combinations, areas that have a decreasing trend are allocated to similar trends as expected, which is either the constant or change point trends. The latter can to some extent mimic an overall decreasing trend by the increasing part of the trend having a much smaller slope than the decreasing part of the trend. Unsurprisingly, none of these areas are allocated to

the increasing trend, and these results provides a sense check that the model performs as expected if an important trend is excluded from the model.

The results from part (b) show correct classification probabilities between 76% and 94%, which, while still quite high, are lower than those presented in the main study when all true trends are included in the model. This reduction in performance is not surprising, because not including the linearly decreasing trend as an option in the model causes those areas with a linearly decreasing trend to be misclassified to one of the other trends (constant or change point). This incorrect classification leads to incorrect parameter estimates for $(\boldsymbol{\beta}, \boldsymbol{\gamma})$, leading to areas being misclassified as the estimated trends do not have the correct magnitude. Note, that for allocation mechanism (B) all areas have linearly decreasing trends, and hence there are no areas to consider in part (b) of the table.

Table 4: The results from omitting the linearly decreasing trend when fitting the model. Section (a) summarises which trends the areas with a true decreasing trend were assigned to. Section (b) summarises the overall correct classification percentages for areas whose true trend was included in the model.

**Scenario**	**(a) Decreasing trend areas classified to**			**(b) Correct classification**
	constant	Increasing	change point	
**(i) A**	76.59%	0%	23.41%	76.06%
**(i) B**	0%	0%	100%	-
**(i) C**	91.61%	0%	8.39%	88.25%
**(ii) A**	81.35%	0%	18.65%	76.76%
**(ii) B**	0.03%	0%	99.97%	-
**(ii) C**	94.15%	0%	5.85%	88.64%
**(iii) A**	69.46%	0%	30.54%	82.50%
**(iii) B**	0.08%	0%	99.92%	-
**(iii) C**	93.96%	0%	6.04%	94.21%
**(iv) A**	79.89%	0%	20.11%	82.72%
**(iv) B**	0.10%	0%	99.9%	-
**(iv) C**	97.2%	0%	2.80%	91.85%

Appendix E - Additional results for the case studies

E.1 - Measles susceptibility case study

Table 5 displays the trend allocation for each of the 1235 intermediate zones in Scotland for models (A) to (C), which have change points in 2002, 2004 and 2006 respectively. There is very little difference in these trend allocations, with almost all of the areas being classified to the change point trend. Furthermore, we refitted the model with hyperparameter values of $a = b = 0.001$ in the inverse-gamma prior for τ^2, and the results again showed little change.

Table 5: Sensitivity analysis of the trend allocations from changing both the year of the change point and the hyperparameters in the inverse-gamma prior for τ^2.

**Hyperparameters**	**Changepoint**	**Temporal trend**			
		Constant	Decreasing	Increasing	Changepoint
$a = 1, b = 0.1$	2002	0	0	0	1235
	2004	2	0	0	1233
	2006	4	1	0	1230
$a = b = 0.001$	2002	0	0	0	1235
	2004	2	0	0	1233
	2006	4	1	0	1230

E.2 - Respiratory hospitalisation case study

Table 6 displays the trend allocations for each of the intermediate zones in the Greater Glasgow and Clyde health board for the linear (A) and monotonic ((B) - 1 knot and (C) - 2 knots) increasing and decreasing trend models. The table shows very similar numbers of IZs allocated to each trend from each model, which shows the results are robust to this choice. Finally, the results from the area specific linear time trends model similar to that proposed by Bernardinelli *and others* (1995), and described in Section 3.4, is compared against our clustering model (3.1) in Figure 6. The figure displays boxplots of the posterior medians of the area specific temporal slope parameters $\{\delta_k\}$ grouped by which trend (decreasing, constant, increasing) model (3.1) assigned the area to. The figure shows that the two models are largely in agreement, in the sense that areas allocated to the decreasing trend have negative δ_k estimates, where as areas allocated to

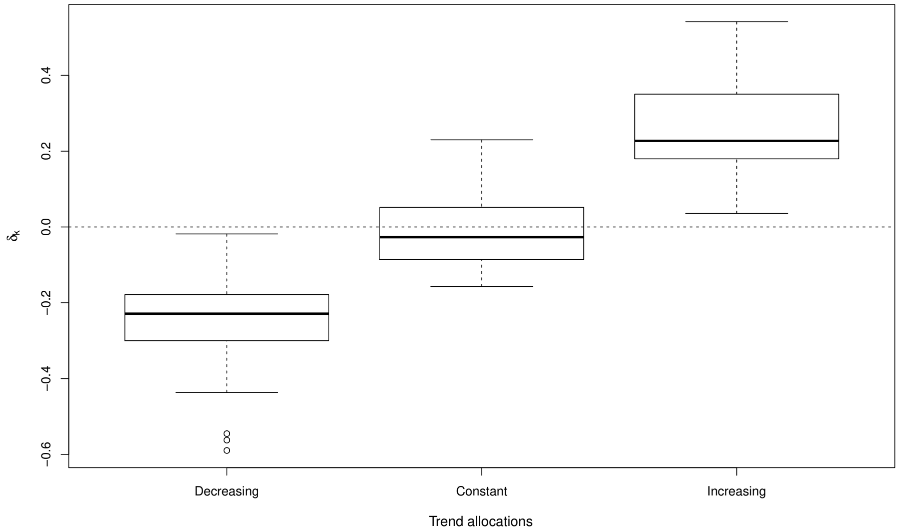

Fig. 6: Boxplots displaying the estimated $\{\delta_k\}$ linear slope estimates grouped by which trend (decreasing, constant, increasing) model (3.1) assigned the area to.

the increasing trend have positive δ_k estimates.

Table 6: Sensitivity analysis of the trend allocations from using linear and non-linear temporal trends and hyperparameters in the inverse-gamma prior for τ^2.

**Hyperparameters**	**Model**	**Temporal trend**		
		Constant	Decreasing	Increasing
$a = 1, b = 0.1$	Linear	122	73	76
	Monotonic (1)	126	71	74
	Monotonic (2)	127	69	75
$a = b = 0.001$	Linear	123	73	75
	Monotonic (1)	122	74	75
	Monotonic (2)	128	68	75

References

Altekar, G., Dwarkadas, S., Huelsenbeck, J. P. and Ronquist, F. (2004). Parallel Metropolis coupled Markov chain Monte Carlo for Bayesian phylogenetic inference. *Bioinformatics* **20**(3), 407–415.

Atchadé, Y. F., Roberts, G. O. and Rosenthal, J. S. (2011). Towards optimal scaling of Metropolis-coupled Markov chain Monte Carlo. *Statistics and Computing* **21**(4), 555–568.

Bernardinelli, L., Clayton, D., Pascuto, C., Montomoli, C., Ghislandi, M. and Songini, M. (1995). Bayesian analysis of space-time variation in disease risk. *Statistics in Medicine* **14**(21–22), 2433–2443.

[*Received August 1, 2010; revised October 1, 2010; accepted for publication November 1, 2010*]
